# Immune checkpoint inhibitors use in lung transplant recipients: a case series and systematic review of literature

**DOI:** 10.1016/j.esmoop.2025.104537

**Published:** 2025-03-31

**Authors:** N. Mahmoud, G. Pamart, C. Nardin, A. Schuller, S. Hirschi, T. Dégot, P.-E. Falcoz, A. Olland, C.-A. Tacquard, R. Kessler, B. Coiffard, B. Renaud-Picard

**Affiliations:** 1 Pulmonology Department, Hôpitaux Universitaires de Strasbourg, Strasbourg, France; 2 Dermatology Department, Université de Franche-Comté, CHU de Besançon, INSERM U1098, Besançon, France; 3 Thoracic Surgery Department, Hôpitaux Universitaires de Strasbourg, Strasbourg, France; 4 Anesthesia and Life Support Department of Nouvel Hôpital Civil, Hôpitaux Universitaires de Strasbourg, Strasbourg, France; 5 Pulmonology Department, CHU de Marseille, Marseille, France

**Keywords:** lung transplant, cancer, immune checkpoint inhibitors

## Abstract

Immune checkpoint inhibitors (ICIs) are an innovative treatment that has improved long-term survival in several neoplastic diseases over the past decade. Solid organ transplant (SOT) recipients, particularly lung transplant (LTx) recipients, have been largely excluded from clinical trials evaluating the safety and efficiency of ICIs, because of the perceived high risk of allograft rejection. In this study, we sought to evaluate the use of ICIs for all neoplastic diseases in LTx patients in all French LTx centers and two Belgian centers. We found only a limited number of cases in which ICIs were suggested to two patients due to a lack of alternative treatments. In the first case, acute respiratory failure and death occurred, whereas in the second case, ICI treatment was well tolerated and resulted in a partial response. In addition, we presented the case of a third LTx patient in whom the use of ICIs was considered but not used due to the patient’s comorbidities. This last case highlights the difficulty of discussing the risk–benefit balance, which ultimately did not favor ICI treatment of this patient. Further multicenter randomized controlled trials are necessary to investigate the safety and efficacy of ICIs in LTx recipients.

## Highlights

• ICI use in lung transplant (LTx) patients across all French and two Belgian LTx centers was evaluated.
• Only two cases were reported, confirming the struggle to use this kind of therapy in these patients.
• Literature on current knowledge about the use of ICI in solid organ transplant patients is presented.

## Introduction

Malignancy is a significant limitation to long-term survival after lung transplantation (LTx), with chronic lung allograft dysfunction (CLAD).^1^ Immune checkpoint inhibitors (ICIs) with antiprogrammed cell death (-ligand) protein 1 [anti-PD-(L)1] and anticytotoxic T-lymphocyte-associated protein 4 (CTLA-4) antibodies are among the most innovative oncology treatments that have improved long-term survival of patients with cancer over the past decade.^2^

Solid organ transplant (SOT) recipients have been largely excluded from clinical trials evaluating the safety and efficiency of ICIs. A few studies have reported data on SOT patients treated with ICIs, showing that one of the major complications is acute allograft rejection (AR).^3^, ^4^, ^5^, ^6^, ^7^, ^8^ The incidence of AR after transplant varies from 25% to 43%, with a higher risk in LTx patients.^5^^,^^9^ The number of studies reporting on LTx patients treated with ICIs remains limited. Consensus guidelines are lacking.^3^^,^^6^^,^^10^

In this study, we asked all 11 LTx French centers and two Belgian centers responsible for ∼3000 LTx patients about their experience with ICI use. Only two cases were reported (Table 1). In addition, we wanted to show how this limited experience influenced our therapeutic strategy for a third LTx patient with skin cancer.

**Table 1:** Summary of reported cases of lung transplant patients treated with checkpoint inhibitors

	Tsung et al.^3^	Daud et al.^10^		Our study	
		Patient #1	Patient #2	Case #1	Case #2
Sex (M/F)	M	F	M	M	M
Age (years)	71	35	72	70	58
Post-LTx main comorbidities	NA	NA	NA	Atrial fibrillation, diabetes, CLAD 6 years after LTx	Primary graft dysfunction, high blood pressure, chronic kidney failure, and an acute rejection episode 2 years after LTx, successfully treated with steroid pulse.
Age at LTx (years)	NA	31	58	63	49
Age at cancer diagnosis (years)	NA	NA	NA	67	58
Cancer type	sSCC	sSCC	Melanoma	sSCC	sSCC
Previous episode of acute rejection (yes/no)	NA	NA	NA	No	Yes
IS treatments before cancer diagnosis	Tacrolimus, prednisolone	Tacrolimus, MMF, prednisolone	Tacrolimus, prednisolone	Tacrolimus, everolimus, prednisolone	Tacrolimus, MMF, prednisolone
IS modification at cancer diagnosis	NA	NA	NA	Stop tacrolimus	Switch from MMF to everolimus
ICI drug	Cemiplimab	Pembrolizumab	Ipilimumab	Cemiplimab	Pembrolizumab
Time from cancer diagnosis to ICI onset (months)	NA	NA	NA	30	5
Acute allograft rejection after ICI (yes/no)	No	No	Yes	Not confirmed	Yes
Time from ICI onset to side-effect onset (months)	NA	NA	12	1	9
Outcome	Immune-mediated pneumonitis.	Chronic allograft dysfunction. Death after 1 year.	Acute allograft dysfunction. Death after 1 year.	Acute allograft dysfunction? Immune-mediated pneumonitis? Death a few days after symptoms onset.	Good response. Untreated mild acute rejection episode. Last news 11 months after ICI onset: still alive.

CLAD, chronic lung allograft dysfunction; F, female; ICI, immune checkpoint inhibitor; IS, immunosuppression; LTx, lung transplantation; M, male; MMF, mycophenolate mofetil; NA, not applicable; sSCC, skin squamous cell carcinoma.

### Case #1

The first patient was a 70-year-old man. He underwent a double LTx for end-stage chronic obstructive pulmonary disease in 2013. In November 2017, he was diagnosed with zygomatic cutaneous squamous cell carcinoma (cSCC). Initial treatment consisted of surgical resection followed by local radiotherapy.

**Figure 1:**
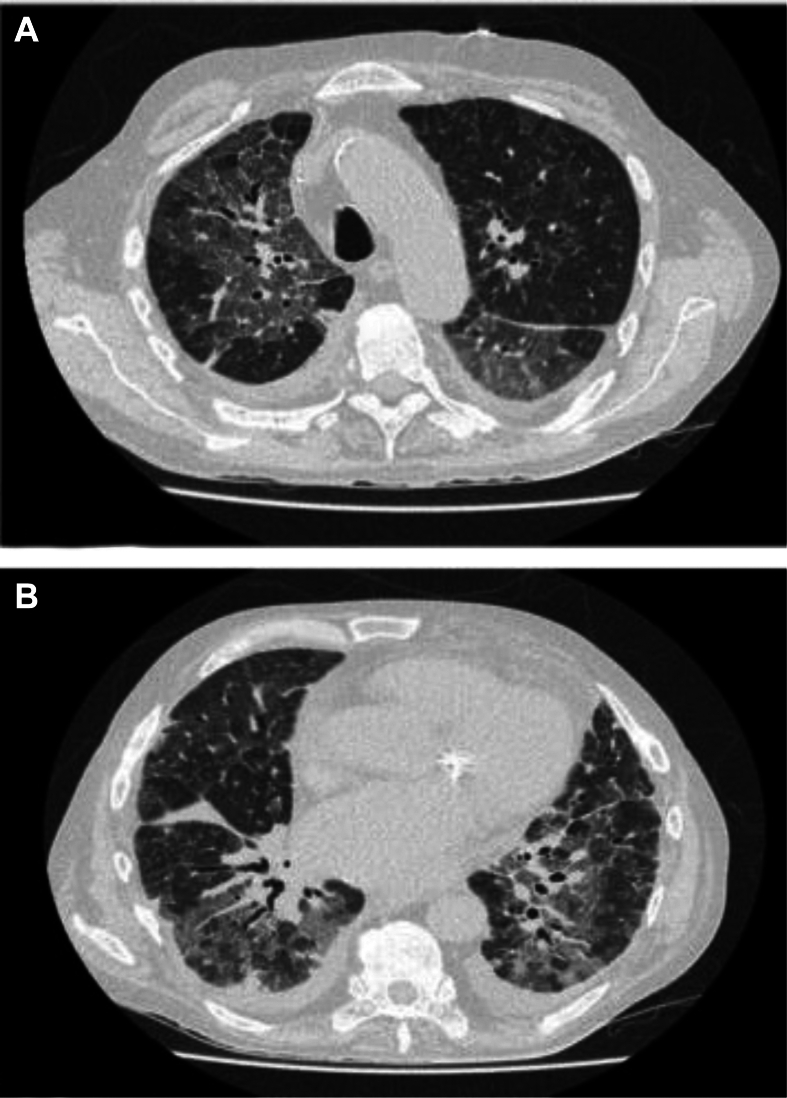
Chest computed tomography scan from Case 1 at the time of the episode of acute respiratory failure after immune checkpoint inhibitor initiation, showing diffuse interstitial lung opacities, with ground-glass opacities (A) and low-volume bilateral pleural effusion (B).

In October 2019, hepatic metastatic recurrence of the cSCC was detected. Tacrolimus was then discontinued, and the patient was treated with combined carboplatin and cetuximab, an anti-epidermal growth factor receptor (EGFR) monoclonal antibody. However, a grade 3 anaphylactic reaction occurred after the first course, including chills, coldness, pallor, pharyngeal paresthesia, and desaturation. Treatment was switched to carboplatin, 5-fluorouracil, and panitumumab, a recombinant humanized anti-EGFR monoclonal antibody, with an initial partial response but poor tolerability and cutaneous and hematological toxicity. The [^18^F]2-fluoro-2-deoxy-D-glucose–positron emission tomography (PET)–computed tomography (CT) scan carried out in April 2020 showed liver and lymph node metastases. After a multidisciplinary consultation regarding the benefit/risk balance, an anti-PD-1 therapy was initiated despite the lack of guidelines for SOT patients. He received the first course of cemiplimab on 1 May 2020. A few days later, the patient developed acute respiratory failure. Viral and bacterial samples were negative. The CT scan showed bilateral multifocal infiltrates and diffuse ground-glass opacities (Figure 1). B-type natriuretic peptide was elevated at 5700 ng/ml (normal range: <400 ng/ml), as was troponin (40 pg/ml—twice the normal range). Left ventricular ejection fraction was 50%, with normal ventricular filling pressures on echocardiography. Myocardial scintigraphy was clinically and electrically normal. Acute ischemic heart disease was excluded, but acute myocarditis was still suspected. Unfortunately, due to the patient’s poor respiratory tolerance, bronchoscopy or invasive investigations were not carried out. Anti-human leukocyte antigen antibodies were negative. The two main diagnostic hypotheses were AR and immune-related adverse events with interstitial lung disease and myocarditis due to ICI.

Cemiplimab was discontinued and the patient was given a steroid pulse to treat a possible AR, followed by abatacept for a possible ICI-induced side-effect. Unfortunately, the patient’s condition worsened and rapidly led to his death.

### Case #2

The second patient was a 58-year-old man who underwent a bilateral LTx at the age of 49 for end-stage pulmonary fibrosis secondary to Sjögren syndrome.

Nine years after LTx, the patient was diagnosed with scalp cSCC with cervical lymph node metastases. Mycophenolate mofetil was subsequently replaced by everolimus. First-line treatment with cetuximab was started in June 2022. Three months later, however, tumor progression was observed in both the scalp and lymph nodes. Surgical resection and radiotherapy were then carried out. After multidisciplinary discussions and in the absence of other therapeutic options at that time, the patient accepted the proposed ICI therapy. Treatment with pembrolizumab, an anti-PD-1 therapy, was initiated in October 2022 and resulted in a favorable tumor response. The treatment was clinically well tolerated, with a mild episode of AR 9 months after the start of ICI therapy. The last dose of pembrolizumab was administered in August 2023. No disease recurrences have been reported during subsequent follow-up.

### Case #3

We present a third 69-year-old male patient who underwent a single LTx in January 2016 for alpha-1 antitrypsin deficiency emphysema. He developed CLAD, diagnosed in October 2016.

In September 2017, he was diagnosed with an NF1-mutated (non-BRAF mutated) melanoma of the cheek with a Breslow thickness of 1.6 mm and without ulceration, for which he successfully underwent surgical excision.

In May 2022, the patient was admitted to hospital with acute respiratory failure and suspected hypoxic infectious pneumonia or AR, for which he was treated with oxygen therapy, broad-spectrum antibiotics, and a high-dose steroid pulse, although histological confirmation could not be obtained as the patient was too fragile to undergo bronchoscopy. Hepatic and cervical lymph node metastases were diagnosed and histologically confirmed. Mycophenolic acid was replaced with everolimus. ICI treatment was discussed but rejected after multidisciplinary discussion, due to the high risk of side effects in this frail, single-LTx patient with CLAD. This decision was made after discussion and agreement with the patient and his wife. Anti-mitogen-activated protein kinase (MEK)-targeted therapy with trametinib targeting the NF1 mutation was started in the absence of other standard treatment options. Initial assessment showed a partial response with poor tolerability, including diarrhea and a decrease in glomerular filtration rate. In October 2022, the patient presented with liver tumor progression. Palliative care was initiated and the patient died a few weeks later.

**Figure 2:**
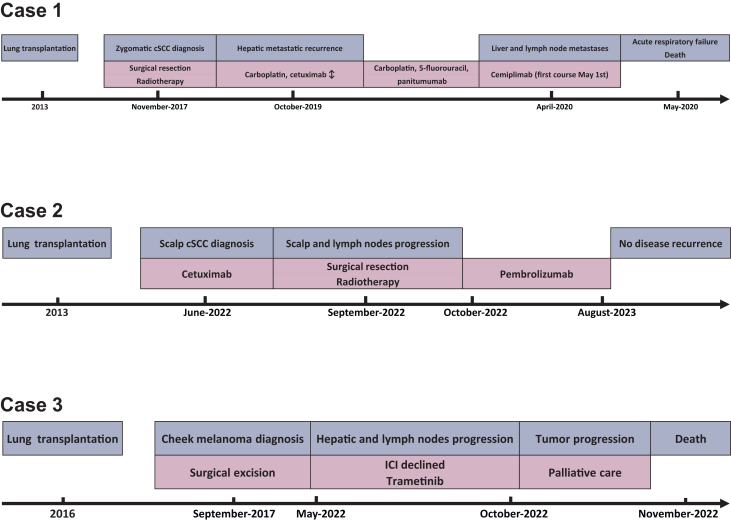
**Summary of the course of history and treatment timeline for all three cases.** cSCC, cutaneous squamous cell carcinoma; ICI, immune checkpoint inhibitor.

All the cases are summarized in Figure 2.

## Discussion

Neoplastic disease is considered one of the major causes of death after LTx, with 17.3% of LTx recipients dying of malignancies 5-10 years after transplantation.^1^ In our own experience, we found that the median survival was higher in LTx recipients without lung cancer (8.77 ± 0.74 years versus 6.19 ± 1.4 years, *P* = 0.042).^11^ In a retrospective study of 308 LTx recipients, 32 patients were diagnosed with a post-transplant malignancy.^12^ Lung cancer was the most common tumor (37%), followed by nonmelanoma skin cancer in six patients (17%), and proliferative disease in four patients (12.5%).

ICIs target inhibitory receptors, known as checkpoints, present on the surface of lymphocytes (CTLA-4, PD-1) or their ligands (PD-L1) on the surface of tumor cells to prevent tumor escape, activate the antitumor response, and induce tumor regression.^13^ ICIs can induce immune-related adverse events of varying severity and frequency depending on the cancer and ICI (anti-PD-1 monotherapy or in combination with anti-CTLA-4).^14^

SOT recipients on long-term immunosuppression (IS) treatment have been largely excluded from all clinical trials investigating the efficiency and safety of ICIs.^15^ One reason is that alloreactive T cells activated by ICIs can lead to AR, a major complication after SOT.^16^ Acute cellular rejection after LTx is mediated by the adaptive immune system, mainly alloantigen-recognizing T cells.^9^ The primary mechanisms leading to donor tissue damage and injury are cytotoxicity and hypersensitivity.^9^^,^^17^ Only a limited number of studies have evaluated the use of ICI therapy in SOT patients.

A first study focused on kidney transplant recipients (KTRs) with cutaneous cancer treated between 2010 and 2020.^5^ An objective response rate (ORR) was observed in 34% of patients. Of 69 KTRs, 42% experienced kidney AR. The median time between the start of ICI therapy and occurrence of AR was 24 days (20-56 days). Factors associated with a lower risk of rejection were the use of mTOR inhibitors [odds ratio (OR) 0.26, 95% CI 0.09-0.72] and triple-agent IS (OR 0.67, 95% CI 0.48-0.92). More recently, real-world data on the use of ICIs, involving 31 KTRs and 1 LTx patient with solid tumors, showed an ORR of 45.2% with a 6-month progression-free survival of 56.8%, and an AR rate of 25.8%, with similar response regardless of the ICI therapeutic strategy used.^6^

The use of ICIs has also been proposed for some liver transplant recipients, particularly for recipients who develop hepatocellular carcinoma (HCC), which is a major problem after liver transplantation. The combination of ICIs and anti-angiogenic drugs is now considered the gold standard for first-line treatment of metastatic HCC in the general population. Kawashima et al.^18^ collected data from 42 liver transplant recipients who received ICI treatment for HCC either before (G1) or after (G2) transplantation. In the G1 group, 25% of patients experienced AR early after transplantation and 30% displayed a response to treatment. In the G2 group, 22.7% of patients experienced AR and 27.3% had a tumor response, with no statistically significant difference between the groups.

Very few studies have described the use of ICIs in heart transplant patients. One study reported nine cases in which patients were treated with ICIs for melanoma (67%), lung adenocarcinoma (11%), lung squamous cell carcinoma (11%), and SCC (11%), of whom 44% had ICI-associated myocarditis, a rare side-effect of ICIs.^18^

Only two previous studies have specifically reported on the use of ICIs in LTx recipients (Table 1). This treatment is undoubtedly challenging, since the AR rate is significantly higher in LTx patients than in other SOT recipients, leading to the use of higher doses of IS maintenance regimens, with no alternative therapeutic option such as dialysis for KTR in the event of terminal rejection.^19^

One of the main questions is in the management of IS maintenance therapy. A first phase 1 trial aimed to assess the risk of AR in 17 KTRs with ICI exposure while maintaining their baseline IS.^4^ No patients had irreversible AR without evidence of tumor response, and there were no treatment-related deaths or serious adverse events. The authors concluded that maintaining baseline IS prior to treatment with an ICI in KTR may not compromise the expected efficacy of the ICI and may reduce the risk of AR. A second phase I study evaluated the use of cemiplimab in 12 KTRs with advanced cSCC.^7^ IS maintenance was switched to an mTOR inhibitor and corticosteroid-specific protocol for each patient. No renal rejection or loss was observed and a response to cemiplimab was observed in 5/11 evaluable patients, suggesting that this IS protocol may represent a favorable strategy for KTRs receiving immunotherapy. Different strategies have then been proposed, such as starting with an increased dose of 20-40 mg of prednisone daily for 2 weeks, followed by 10 mg for maintenance to prevent AR.^4^^,^^6^, ^7^, ^8^

In our study, the first patient received a suboptimal IS maintenance regimen after LTx, consisting only of steroids and mTOR inhibitor, which are commonly used as both anticancer treatment and IS maintenance regimen in SOT patients, especially after cancer diagnosis.^5^^,^^20^ Transbronchial biopsies were not performed to confirm AR due to the patient’s frailty. In addition, the patient may have had immune-related myocarditis and pneumonitis.^21^ The second patient was still alive after several months of treatment and showed a complete response. His IS maintenance regimen included tacrolimus, which is known to be better at preventing AR, although it did not prevent a mild and well-tolerated episode of AR.^22^ Our third observation involved an elderly and frail patient with a history of CLAD. Despite disease progression after poorly tolerated anti-MEK therapy, the risk associated with ICI was deemed too high and the treatment was not initiated due to the patient’s comorbidities. This case highlights the struggle with LTx patients at higher risk of complications and the importance of discussing each critical situation individually, possibly with the support of multidisciplinary meetings.

In this review, we have summarized the current knowledge on the use of ICI in LTx patients. We have described the global experience of French centers and two Belgian LTx centers in the use of ICIs in LTx patients, with different results, and have confirmed that this is still extremely limited, as LTx centers remain very cautious due to the lack of data and the suspected high risk of AR, which prevents us from drawing strong conclusions from these data. The lack of biopsies performed to document possible AR is another important limitation.

## Conclusion

Previous studies have suggested that ICIs could be considered for SOT patients with cancer if the potential benefits of treatment outweigh the risks of allograft loss and if no other effective cancer treatments are available. There is insufficient evidence to make some recommendations. Further multicenter randomized controlled trials are needed to investigate the safety and efficacy of ICIs in LTx recipients.
