# Supplementary material for: Immune checkpoint inhibitors use in lung transplant recipients: a case series and systematic review of literature
Source: ESMO Open. 2025 Mar 31;10(4):104537. doi: 10.1016/j.esmoop.2025.104537 (PMC11999205; doi:10.1016/j.esmoop.2025.104537)
Supplement: Supplemental Data [file mmc1.docx]

**Benjamin RENAUD-PICARD**

[benjamin.renaudpicard@chru-strasbourg.fr](mailto:benjamin.renaudpicard@chru-strasbourg.fr)

No twitter account to report
